# PML isoforms in response to arsenic: high-resolution analysis of PML body structure and degradation

**DOI:** 10.1242/jcs.132290

**Published:** 2014-01-15

**Authors:** Katherine J. Hands, Delphine Cuchet-Lourenco, Roger D. Everett, Ronald T. Hay

**Affiliations:** 1 Wellcome Trust Centre for Gene Regulation and Expression, College of Life Sciences, University of Dundee, Dundee DD1 5EH, UK; 2 MRC, University of Glasgow Centre for Virus Research, Church Street, Glasgow G11 5JR, UK

**Keywords:** PML, Arsenic, SUMO, RNF4

## Abstract

Arsenic is a clinically effective treatment for acute promyelocytic leukaemia (APL) in which the promyelocytic leukaemia (PML) protein is fused to retinoic receptor alpha (RARα). PML-RARα is degraded by the proteasome by a SUMO-dependent, ubiquitin-mediated pathway in response to arsenic treatment, curing the disease. Six major PML isoforms are expressed as a result of alternative splicing, each of which encodes a unique C-terminal region. Using a system in which only a single EYFP-linked PML isoform is expressed, we demonstrate that PMLI, PMLII and PMLVI accumulate in the cytoplasm following arsenic treatment, whereas PMLIII, PMLIV and PMLV do not. 3D structured illumination was used to obtain super-resolution images of PML bodies, revealing spherical shells of PML along with associated SUMO. Arsenic treatment results in dramatic isoform-specific changes to PML body ultrastructure. After extended arsenic treatment most PML isoforms are degraded, leaving SUMO at the core of the nuclear bodies. A high-content imaging assay identifies PMLV as the isoform most readily degraded following arsenic treatment, and PMLIV as relatively resistant to degradation. Immunoprecipitation analysis demonstrates that all PML isoforms are modified by SUMO and ubiquitin after arsenic treatment, and by using siRNA, we demonstrate that arsenic-induced degradation of all PML isoforms is dependent on the ubiquitin E3 ligase RNF4. Intriguingly, depletion of RNF4 results in marked accumulation of PMLV, suggesting that this isoform is an optimal substrate for RNF4. Thus the variable C-terminal domain influences the rate and location of degradation of PML isoforms following arsenic treatment.

## INTRODUCTION

The promyelocytic leukaemia (PML) protein was first identified as part of the t(15∶17) chromosomal translocation found in patients with the disease acute promyelocytic leukaemia (APL) (de Thé et al., 1990) where it is fused to the retinoic receptor alpha (RARα). PML is a member of the tripartite motif (TRIM) family of proteins, and contains a RING domain and two B boxes, all of which are zinc-binding domains, and a coiled-coil domain (Jensen et al., 2001), which is important for interactions between PML molecules. Alternative splicing of the PML mRNA transcript results in the expression of seven major PML isoforms, six of which are predominantly nuclear (Jensen et al., 2001). These different isoforms share a common N-terminal region, encoded by the first six exons, which contains all components of the TRIM, but differ in their C-terminal parts because of expression of various combinations of exons seven to nine (Jensen et al., 2001). These varying C-terminal parts are thought to be responsible for isoform-specific interactions (Condemine et al., 2007; Fogal et al., 2000; Xu et al., 2005; Yu et al., 2010) and functions (Cuchet et al., 2011).

A distinguishing feature of PML is that it is found in discrete subnuclear structures known as PML nuclear bodies (PML-NBs). PML is modified by the small ubiquitin-like modifier (SUMO) on three lysine residues, K65, K160 and K490 (Kamitani et al., 1998), located within the N-terminal regions common to all isoforms. PMLI and PMLIV are also modified on K616, a lysine residue encoded by exon 8a, common to only these two isoforms (Cuchet-Lourenço et al., 2011). SUMO modification of PML is required for the formation of normal PML-NBs (Ishov et al., 1999). As many as 70 proteins have been reported to localise to PML-NBs (Negorev and Maul, 2001), and PML-NBs have been implicated in the regulation of multiple cellular processes, including the antiviral response, transcription, apoptosis and DNA repair (Bernardi and Pandolfi, 2007; Lallemand-Breitenbach and de Thé, 2010).

Arsenic trioxide has been used for many years as an extremely effective treatment for APL (Hu et al., 2009; Mathews et al., 2010; Powell et al., 2010); however, its mechanism of action has only recently been elucidated. Following arsenic treatment, PML is recruited from the nucleoplasm to PML-NBs and rapidly modified with poly-SUMO chains (Lallemand-Breitenbach et al., 2001; Zhu et al., 1997). Subsequently, the SUMO-targeting ubiquitin E3 ligase RNF4 is recruited to PML-NBs in a SUMO-dependent manner (Geoffroy et al., 2010), where it ubiquitylates poly-SUMO chains present on PML, targeting PML for degradation by the proteasome (Geoffroy et al., 2010; Lallemand-Breitenbach et al., 2008; Tatham et al., 2008).

Previous reports of the specific characteristics and functions of individual PML isoforms have primarily used systems in which a single isoform is overexpressed on a background of endogenous PML (Beech et al., 2005; Maroui et al., 2012; Weidtkamp-Peters et al., 2008). PML isoforms interact with each other (Condemine et al., 2006), and therefore studies investigating the characteristics of these overexpressed isoforms might be confounded by the fact that the overexpressed isoform will interact with endogenous PML. Others have used isoform-specific antibodies to investigate the properties of the various endogenous PML isoforms (Condemine et al., 2006). Here we use a system in which a single PML isoform is stably expressed at close to endogenous levels in cells in which expression of endogenous PML was depleted by the stable expression of a short-hairpin RNA (Cuchet et al., 2011) to assess the effects of arsenic treatment on each of the six main PML isoforms. High-resolution microscopy was used to follow changes in PML body ultrastructure in response to arsenic. Biochemical analysis identified PMLV as the isoform most readily degraded in response to arsenic treatment, because it is the most heavily SUMO modified and therefore an optimal substrate for RNF4.

## RESULTS

### PML isoforms degrade at different rates following arsenic treatment

**Fig. 1.**
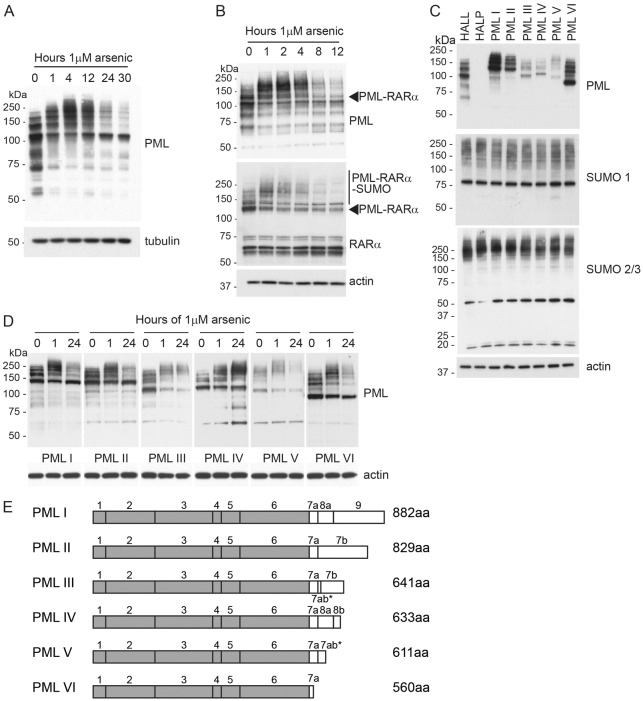
**Western blot analysis of response of PML to arsenic treatment.** (A) HepaRG hepatocytes were treated with 1 µM arsenic trioxide for the indicated periods of time before lysis. Whole-cell extracts were then analysed by SDS-PAGE and immunoblotting with chicken anti-PML antibody and mouse anti-tubulin antibodies. (B) The NB4 leukaemia cell line which expresses the PML-RARα fusion was treated with arsenic for the indicated periods of time before lysis. Cell extracts were then analysed by SDS-PAGE and immunoblotting with chicken anti-PML, rabbit anti-RARα and mouse anti-actin antibodies. Arrowheads indicate the PML-RARα fusion protein. (C) HepaRG hepatocytes stably expressing a control short hairpin RNA (shRNA), denoted HALL, stably expressing an anti-PML shRNA, HALP, or stably expressing both the anti-PML shRNA plus EYFP-PML constructs, PMLI–PMLVI, were lysed and extracts analysed by western blotting with antibodies specific for PML, SUMO1 and SUMO2/3 and actin. Species identified by anti-PML antibody in HALL cells are endogenous PML, whereas exogenous constructs are detected with PMLI–PMLVI. (D) HepaRG hepatocytes depleted of endogenous PML which express EYFP-PML isoforms I–VI were treated with 1 µM arsenic for the indicated periods of time prior to lysis. Whole-cell extracts were analysed by SDS-PAGE and immunoblotting with a chicken anti-PML and mouse anti-actin antibodies. (E) Schematic representation of the exon structure of the six nuclear PML isoforms. All isoforms encode exons 1–6, but alternative splicing of exons 7–9 result in varying C-termini as shown. The size of the translated product is shown on the right. Asterisk indicates a retained intron.

It is now well established that PML undergoes SUMO-dependent ubiquitin-mediated proteolysis in response to arsenic trioxide treatment (Lallemand-Breitenbach et al., 2008; Tatham et al., 2008; Weisshaar et al., 2008). When assessed by western blotting, PML was identified as multiple species, which represent isoforms encoded by differentially spliced mRNAs and their post-translationally modified forms (Fig. 1A). Following arsenic treatment at a therapeutically relevant concentration, PML rapidly accumulated as higher molecular weight species. Previous work has demonstrated this to represent post translational modification of PML with SUMO and subsequently ubiquitin. At later time points, total PML species were reduced, owing to their proteasomal degradation. However, some species of PML persist (Fig. 1A), indicating that not all forms of PML are degraded at the same rate in response to arsenic treatment.

The PML-RARα fusion product in acute promyelocytic leukaemia represents a unique PML isoform in which PML is present at the N-terminus, with RARα forming the C-terminus of the oncoprotein. We treated NB4 leukaemia cells that contain the PML-RARAα fusion with arsenic trioxide and compared the degradation of PML-RARα with that of endogenous PML (Fig. 1B). Western blotting of whole-cell extracts with antibodies specific for PML and RARα demonstrate that the PML-RARα fusion is post-translationally modified and degraded at a similar rate to endogenous PML, suggesting the introduction of the RARα at the C-terminus does not impair the ability of PML to be SUMO modified or inhibit the action of the ubiquitin E3 ligase RNF4.

### Cells expressing a single PML isoform

To assess the role of the variable C-terminal regions of PML isoforms in determining response to arsenic treatment, we used a system in which a single EYFP-linked PML isoform is stably expressed in HepaRG hepatocytes in which endogenous PML has been stably depleted by expression of an anti-PML short-hairpin RNA (Cuchet et al., 2011). This system allows the true response of a single PML isoform to be assessed in isolation by removing possible interactions between isoforms that are demonstrated to take place in systems where a single isoform is overexpressed on a background of endogenous PML. We can thus assess the response to arsenic of the six major PML isoforms, PMLI–PMLVI, using these cell lines. Western blotting of cell extracts with an anti-PML antibody revealed a different profile of bands for each isoform, representing the PML isoform and its various post-translationally modified forms (Fig. 1C, top panel). The expression levels of the different PML isoforms varied in these cells (supplementary material Fig. S1A), as did the levels of the endogenous PML isoforms previously examined in various cell lines (Condemine et al., 2006). These apparent differences in expression reflect the varying degrees of post-translational modification of each isoform, as well as the relative stability of the isoforms under basal conditions. The profile of total SUMO1 conjugates does not differ significantly between cell lines, although there might be slight variations in the patterns of overall SUMO2/3 conjugates in the different cell lines (Fig. 1C).

To assess the response of the different PML isoforms to arsenic treatment, each cell line was exposed to arsenic for various periods of time, and the resulting whole-cell extracts analysed by western blotting with an anti-PML antibody (Fig. 1D). As anticipated, differences in the response to arsenic were identified. All isoforms appeared to undergo additional post-translational modification in response to arsenic, as demonstrated by a change in electrophoretic mobility of PML observed after 1 hour of treatment, with the appearance of higher molecular weight PML species. After 24 hours of treatment, there was extensive loss of these modified forms in most cases, but some PML isoforms had been degraded more than others (supplementary material Fig. S1B). Treatment of PMLIV with arsenic trioxide resulted in marked accumulation of high molecular weight PML species, but little or no apparent degradation, whereas PMLIII and PMLV were initially SUMO modified before being extensively degraded. PMLI, PMLII and PMLVI showed similar patterns of response: they were initially modified, then these modified species were degraded, but there was a reappearance of a major species that is likely to represent newly synthesised unmodified material that accumulates after 24 hours of treatment. Extending the length of arsenic treatment did not affect the patterns of degradation seen (data not shown). Although the PML isoforms (Fig. 1E) were expressed at different levels (Fig. 1C), the ability to be degraded did not correlate with the levels of the expressed protein, because PML isoform IV was expressed at low levels (Fig. 1C) and yet was largely resistant to arsenic-induced degradation (Fig. 1D).

### Subcellular localisation of PML isoforms differs following arsenic treatment

**Fig. 2.**
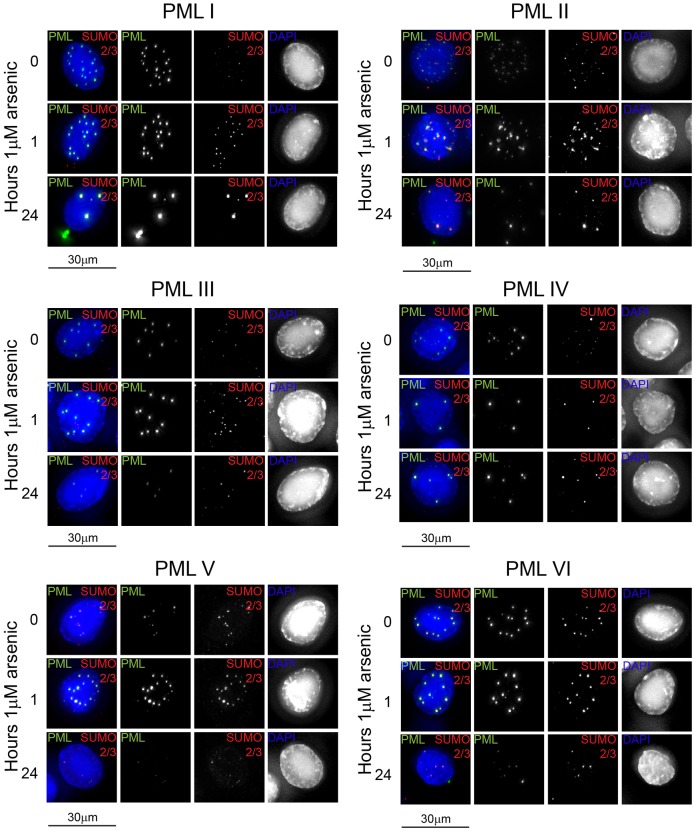
**Immunofluorescence analysis of PML isoforms following arsenic treatment.** Cells expressing a single EYFP-PML isoform were cultured on coverslips and treated with 1 µM arsenic. Cells were fixed at the time points described and immunostained with a sheep anti-SUMO2/3 antibody, fluorescently labelled anti-sheep IgG secondary antibody and DAPI to stain DNA. EYFP-PML fluorescence is shown in green, SUMO2/3 in red. Images are presented as maximal intensity projections of multiple *z*-slices.

By exploiting the fact that these cells express an EYFP-linked PML isoform, deconvolution microscopy was used to evaluate the subcellular localisation of the PML isoforms in response to arsenic treatment. All PML isoforms examined formed punctate structures resembling PML nuclear bodies, demonstrating that different molecules of the same isoform interact in the absence of other isoforms (Fig. 2). Immunofluorescence microscopy performed using an anti-SUMO2/3 antibody revealed that SUMO2/3 colocalises with PML bodies in all cell lines in untreated cells. The initial response of all isoforms following arsenic treatment was similar. Following 1 hour of arsenic treatment, there was an increase in the number of PML bodies present, and an increase in the amount of SUMO2/3 (Fig. 2) and SUMO-1 (data not shown) present at these PML bodies. This is consistent with the findings in Fig. 1D, where an increase in high molecular weight PML species was seen after 1 hour of arsenic treatment. After prolonged arsenic treatment, differences in response become apparent, with PMLI, PMLII and PMLVI exhibiting additional PML foci in the cytoplasm. This cytoplasmic PML following arsenic treatment did not colocalise with SUMO2/3, which suggests that this PML has either been desumoylated or is newly synthesised and has yet to be SUMO modified (Fig. 2). Consistent with the observation in Fig. 1D, PMLIV was not significantly degraded following prolonged arsenic treatment, whereas PMLIII and PMLV were greatly reduced in intensity, consistent with the degradation data of Fig. 2.

### Quantification of fluorescence confirms differing responses to arsenic

**Fig. 3.**
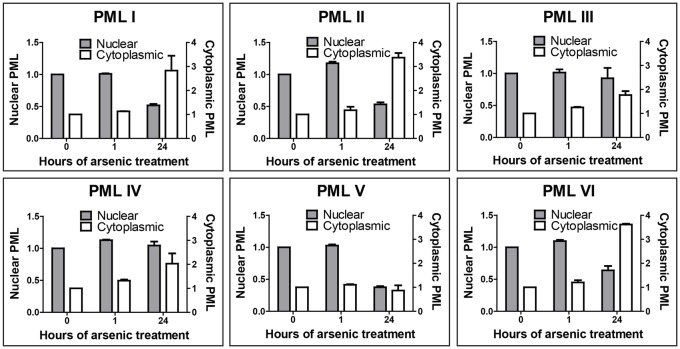
**High-content imaging of PML isoforms and quantification of fluorescence.** High-content imaging was performed of cells expressing a single EYFP-PML isoform. Cells were cultured in 96-well plates, treated with arsenic, fixed with paraformaldehyde and imaged on the automated IN Cell 2000 microscope. PML fluorescence in ∼5000 cells per time point was analysed. Data presented represent the sum of the area of individual PML nuclear bodies, or cytoplasmic PML inclusions per cell, averaged for each PML isoform after 0, 1 or 24 hours of arsenic treatment. Data are normalised to the average total cellular area of nuclear or cytoplasmic PML present in untreated cells. Data represent the mean ± s.e.m.; grey bars, nuclear PML; unfilled bars, cytoplasmic PML.

To quantify the differences identified in the response of the PML isoforms to arsenic, we established a high-content imaging assay to enable automated imaging and analysis of EYFP-PML fluorescence. Cells were cultured in 96-well plates and treated with arsenic for various lengths of time as in the previous experiments. Following fixation, cells were stained with DAPI and imaged using an IN Cell 2000 automated microscope. Analysis of the resulting images was performed using IN Cell Investigator software, with a protocol designed to quantify the size, number and location of EYFP-PML nuclear bodies or cytoplasmic inclusions. This automated approach allowed quantification of PML expression for upwards of 5000 cells per condition. The most discriminatory measure of changes in EYFP-PML expression was found to be the total area of PML within the nucleus or cytoplasm of a given cell, which is the sum of the area of individual PML nuclear bodies or cytoplasmic inclusions. These EYFP-PML bodies and inclusions were identified by the software when pixel clusters have an EYFP intensity that exceeds a given threshold above background. These data (Fig. 3) demonstrate that for PMLI, PMLII and PMLVI, nuclear PML decreases by ∼50 per cent after 24 hours of arsenic treatment. Conversely, and as expected from the data of Fig. 2, PML accumulated in the cytoplasm of these cells, increasing by up to 3.5-fold. PMLV was confirmed to be readily degraded by arsenic treatment, with the amount of nuclear PML decreasing by two-thirds, with no increase in cytoplasmic PML. Considering the data of Fig. 1D, Figs 2 and 3 together, it is possible that the unmodified PMLI, PMLII and PMLVI detected by western blotting of whole-cell extracts after 24 hours of arsenic treatment represents the cytoplasmic fraction of PML, given that cytoplasmic PML did not colocalise with SUMO2/3 by immunofluorescence (Fig. 2). Of note, the PML isoforms did not accumulate in the cytoplasm of these cells treated with both arsenic and the proteasome inhibitor MG132, indicating that proteasomal function is required for this relocalisation to take place (data not shown).

### Super-resolution imaging reveals differences in PML body structure

**Fig. 4.**
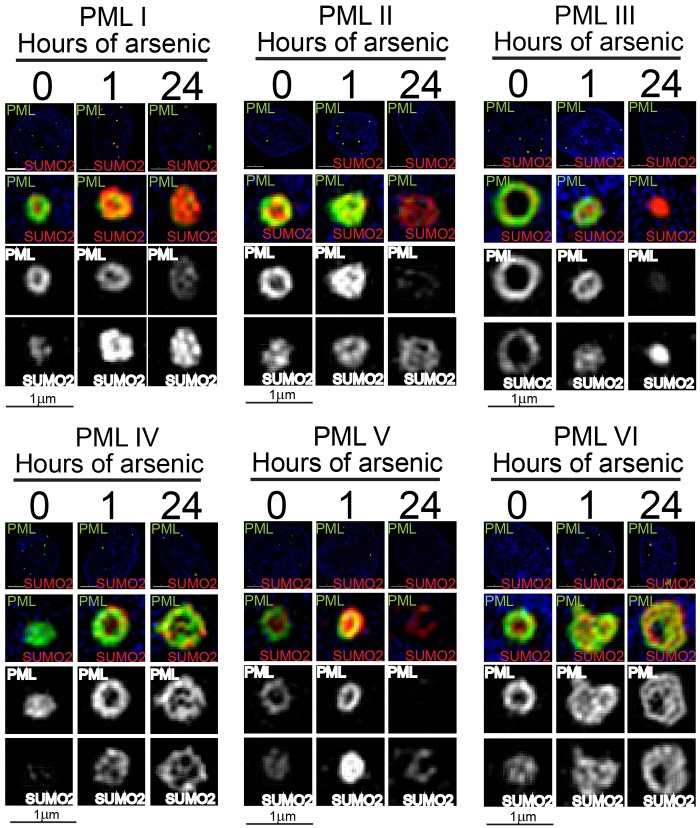
**Super-resolution imaging of PML nuclear body structure.** Cells expressing a single EYFP-PML isoform were imaged using structured illumination to gain high-resolution images of PML nuclear bodies. Cells were fixed with paraformaldehyde and immunostained with an anti-SUMO2/3 antibody and a corresponding anti-sheep IgG fluorescently labelled secondary antibody. DNA was stained with DAPI. Images are a cross-section through the centre of a representative PML nuclear body. PML is shown in green, SUMO2/3 in red.

As demonstrated in Fig. 2 and reported previously (Cuchet et al., 2011), the individually expressed PML isoforms formed nucleate punctate structures that were associated with SUMO modification. Previous electron microscopy studies with immunolabelling revealed that PML bodies are ring shaped (Boisvert et al., 2000; Koken et al., 1994; Lallemand-Breitenbach et al., 2001) and more recent work using high resolution microscopy (Hattersley et al., 2011; Lang et al., 2010) confirmed that PML bodies consist of a spherical shell of PML, with SUMO1 and SUMO2/3 found predominantly interspersed within the PML shell and in the central core of the structure, respectively. We investigated the structure of the PML bodies formed by the individual isoforms and the changes in structure induced by arsenic treatment using three-dimensional structured illumination microscopy (3D SIM) to obtain super-resolution images. The images confirmed that all PML isoforms form nuclear bodies consisting of hollow, near-spherical shells of PML and that SUMO2/3 is associated with these shells (Fig. 4). However, the location of SUMO2/3 in relation to PML differs between isoforms. In untreated cells, SUMO2/3 was found in the central core of PML bodies formed by PMLI, PMLII and PMLV, consistent with previous reports, but SUMO2/3 was incorporated predominantly into the PML outer shell of the nuclear bodies formed by PMLIII. PMLVI nuclear bodies had SUMO2/3 in both locations (Fig. 4). Following 1 hour of arsenic treatment, there was an increase in SUMO2/3 associated with all PML isoforms, but this was particularly marked in cells expressing PMLI and PMLV, where there was accumulation of SUMO2/3 associated with the outer shell of PML, as well as in the central core. The most dramatic changes in nuclear body structure were seen after 24 hours of arsenic treatment. At this point, the nuclear bodies were still visible as essentially spherical structures consisting of PML and SUMO2/3, but the well-defined outer shell of PML was no longer seen in cells expressing PMLI, PMLII, PMLIV or PMLVI. Rather, PML was present both in the outer part of and within the central area of the nuclear body and localised closely with SUMO2/3. The PMLIII bodies appeared to have collapsed into smaller discrete foci in which PML and SUMO2/3 colocalised. Interestingly, in cells expressing PMLV, PML has been efficiently degraded after 24 hours of arsenic treatment but the remnants of PML nuclear bodies could be identified by a spherical hollow shell of SUMO2/3. This might represent either a tiny fraction of very heavily SUMO2/3-modified PML (below the limit of detection of EYFP fluorescence), or SUMO modification of other PML-NB components such as Sp100.

### All PML isoforms are modified with SUMO and ubiquitin following arsenic treatment

**Fig. 5.**
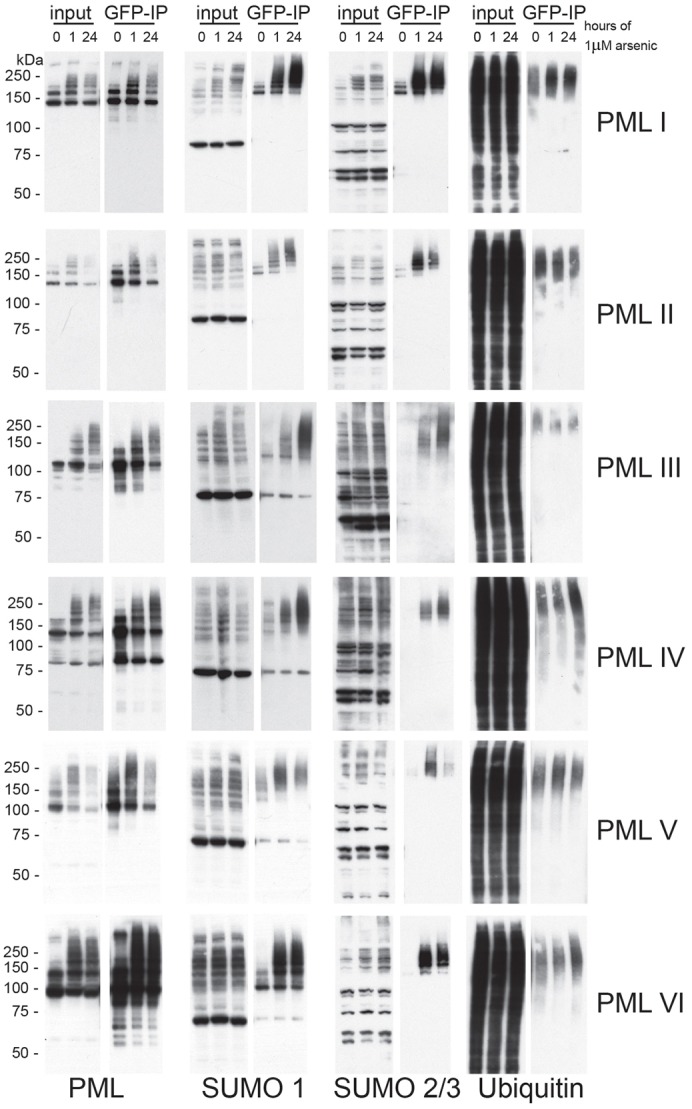
**Characterisation of arsenic-induced post-translational modification of PML.** Cells expressing EYFP-PML isoforms were treated with arsenic and lysed under conditions to maintain SUMO modification. Lysates were then subjected to GFP immunoprecipitation and eluted proteins analysed by SDS-PAGE and immunoblotting with antibodies specific for PML, SUMO1, SUMO2/3 and ubiquitin.

The changes in the appearance of the high molecular weight PML species following arsenic treatment (Fig. 1D) suggest that all PML isoforms tested are post-translationally modified in response to arsenic treatment. Immunofluorescence experiments confirmed an increase in colocalisation of PML and SUMO2/3 following arsenic treatment (Fig. 2), suggesting that each isoform undergoes additional SUMO modification in response to arsenic treatment. Given that it is well established that endogenous PML is rapidly SUMO modified in response to arsenic, and that this SUMO-modified PML is a substrate for the ubiquitin E3 ligase RNF4, we wished to determine whether all isoforms follow this pathway of arsenic induced degradation. To biochemically characterise these modifications following arsenic treatment, we performed immunoprecipitation under stringent conditions of EYFP-PML and its conjugates (using an anti-GFP antibody) from each of the isoform expressing cell lines after varying exposures to arsenic. The data presented in Fig. 5 demonstrate that all isoforms are modified with SUMO1 and SUMO2/3, although significant differences between isoforms were identified. With the exception of PMLI and PMLIV, the recovery of ubiquitylated forms of the various isoforms did not vary greatly during arsenic treatment, perhaps because most ubiquitylated forms will be rapidly degraded. Although it is difficult to compare the amount of SUMO modification between different isoforms owing to differences in PML expression levels, it is possible to analyse the differences in modification for any given isoform. PMLI and PMLII show low levels of SUMO2/3 modification prior to arsenic treatment, whereas little or no SUMO2/3 modification of PMLIII, PMLIV, PMLV and PMLVI is detectable in untreated cells. In untreated cells, SUMO1 and SUMO2/3 modification of PMLI and PMLII manifested as two distinct species, whereas the PMLV species that could be detected appeared as a high molecular weight smear, suggesting it is modified with polymeric SUMO chains. There was a marked increase in SUMO1 modification, and an even more substantial increase in SUMO2/3 modification of PMLI, PMLII and PMLVI following 1 hour of arsenic treatment. This increase in modification does not occur to the same extent with other isoforms, with the most marked modification of PMLIII and PMLIV taking place after 24 hours of arsenic treatment. Given that there was a decrease in the amount of EYFP-PMLI, -PMLII and -PMLIII immunoprecipitated after 24 hours of arsenic treatment, the fact that there was substantially more SUMO1 and SUMO2/3 co-immunoprecipitated suggests that this small amount of PML is extensively SUMO modified. Fig. 3 indicates that PMLIV was relatively stable in the presence of arsenic treatment, however Fig. 5 demonstrates that PMLIV is modified by both SUMO1 and SUMO2/3 in response to arsenic. As noted above, there was no significant decrease in the amount of EYFP-PMLIV immunoprecipitated after 24 hours of treatment, suggesting that it is not significantly degraded. There was also an increase in ubiquitin modification of PMLIV at this time point, suggesting that despite ubiquitin modification, the protein is not being efficiently degraded by the proteasome.

### RNF4 is required for arsenic-mediated degradation of all PML isoforms

**Fig. 6.**
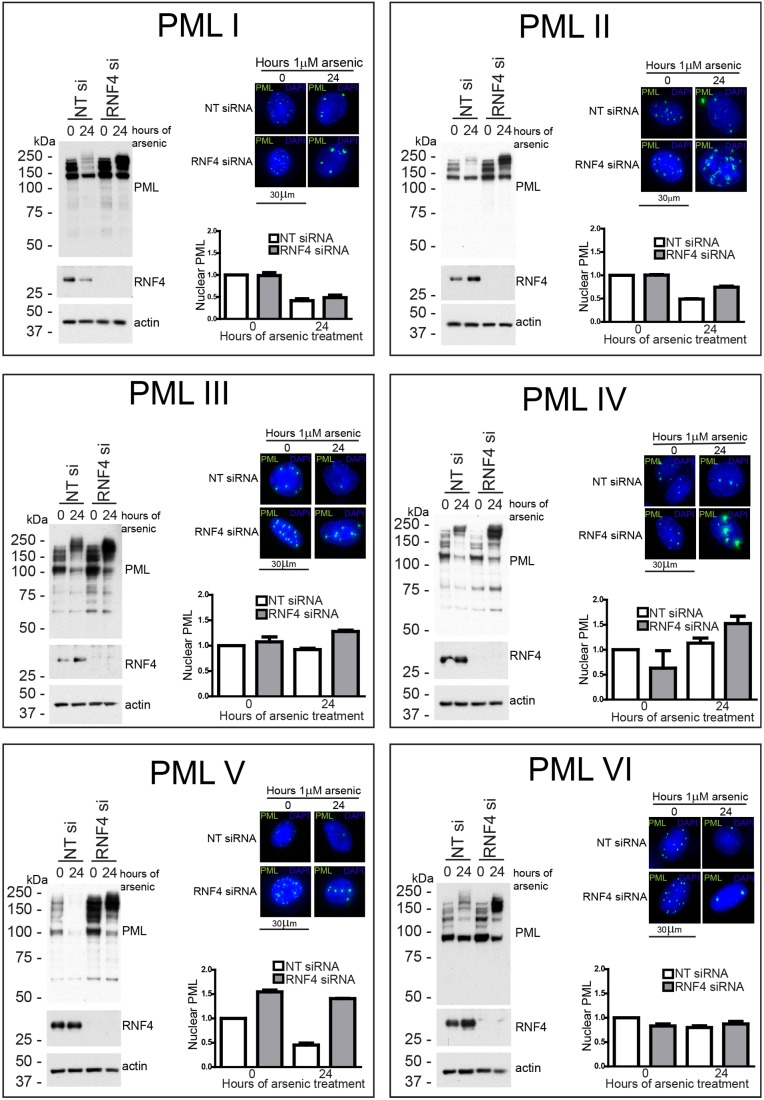
**RNF4 is required for arsenic-mediated degradation of all PML isoforms.** HepaRG cells expressing a single PML isoform were transfected with either non-targeting control siRNA (NT si) or RNF4 siRNA (RNF4 si) and 48 hours later treated with 1 µM arsenic for 24 hours. Cells were lysed for western blotting and fixed with paraformaldehyde for fluorescence microscopy. Western blotting was performed with anti-PML, anti-RNF4 and anti-actin antibodies. Cells for fluorescence microscopy were stained with DAPI. The same experiment was performed in 96-well plates to allow high-content imaging and quantification of EYFP-PML fluorescence as described in Fig. 4. Approximately 5000 cells were analysed for each condition. Graphs represent the sum of the area of all PML nuclear bodies within a given cell nucleus, averaged for all cells examined in the particular cell line at the time point. Data are normalised to non-transfected, non-arsenic-treated cells. Bars represent s.e.m.

Having confirmed that all isoforms are modified by SUMO and ubiquitin in response to arsenic treatment, we wished to investigate the effects of depletion of the SUMO-specific ubiquitin E3 ligase RNF4 on the response of the different isoforms to arsenic. Using a pool of four siRNAs targeting RNF4, we efficiently depleted RNF4 from cells expressing the PML isoform before treatment with arsenic for 24 hours. Depletion of RNF4 was confirmed by western blotting, and the effect on PML assessed by western blotting and fluorescence microscopy (Fig. 6). To quantify the differences observed, the high-content imaging assay used in Fig. 3 was modified such that cells were transfected with siRNA in a 96-well plate format prior to arsenic treatment and imaging as before. Accumulation of high molecular weight PML species was identified for all isoforms following RNF4 depletion and arsenic treatment, suggesting that RNF4 is indeed required for ubiquitylation and subsequent proteasomal degradation of each isoform. The most striking observation was the marked accumulation of PML V following RNF4 depletion alone, indicating that PMLV is a very good substrate for RNF4. This is in agreement with the observation that PMLV was extensively SUMO modified in untreated cells, and a high rate of consequent RNF4-dependent degradation might account for the low expression levels seen in untreated cells. It is also consistent with the observation that PMLV is the most efficiently degraded isoform in response to arsenic treatment.

## DISCUSSION

SUMO-dependent ubiquitin-mediated proteolysis of PML and PML-RARα in response to arsenic treatment is responsible for arsenic-induced cure of acute promyelocytic leukaemia (Lallemand-Breitenbach et al., 2008; Tatham et al., 2008). Six major PML isoforms are expressed as a result of alternative splicing of the primary PML transcript. These isoforms share a common N-terminal region, encoded by exons 1–6, but express unique C-terminal parts. Western blotting of endogenous PML after arsenic treatment revealed PML species that were relatively resistant to arsenic-induced degradation, suggesting that different PML isoforms respond differently to arsenic treatment (Fig. 1A). Although isoform-specific sequences are dispensable for arsenic-induced degradation (Jeanne et al., 2010), they nevertheless influence the rate of PML degradation in response to arsenic. The mechanism by which the isoform-specific protein sequences influence degradation is unclear, but it is likely that they bind factors that either interfere with or enhance the pathway induced by arsenic that leads to degradation. To investigate the role of the various C-terminal regions in the response of individual PML isoforms to arsenic treatment, we treated cell lines expressing only a single EYFP-linked PML isoform with arsenic and identified significant differences in their response to this treatment. PMLIV was particularly resistant to arsenic-induced degradation, whereas PMLV was the most readily degraded (Fig. 1D). This is not entirely consistent with a previous analysis of the response to arsenic of endogenous PML isoforms using isoform-specific antibodies (Condemine et al., 2006), in which all isoforms were found to be degraded. However, the various isoforms in the previous study will form heterodimers with each other. The approach taken in this study allows differences in the response of the individual isoforms to be uncovered. In a recently published study (Maroui et al., 2012) in which individual PML isoforms were expressed on a background of endogenous PML expression, it was demonstrated that the SIM in PML was required for arsenic-induced degradation. However, this is inconsistent with our observation that PML-RARα (Fig. 1B) and PMLVI (Fig. 1D), which both lack the SIM, are degraded in response to arsenic.

To further investigate the structure of PML-NBs, 3D structured illumination microscopy was used to obtain high- resolution images of PML-NBs before and after arsenic treatment. This demonstrated that nuclear bodies formed by all isoforms consist of a spherical shell of PML, and is associated with SUMO2/3. The distribution of SUMO2/3 differed between isoforms. Consistent with previous reports, SUMO2/3 was found in the central core of PML-NBs formed by PMLI, PMLII and PMLV. However SUMO2/3 was incorporated into the outer PML shell of PML-NBs formed by PMLIII. A number of PML isoform-specific interactions have been described (Fogal et al., 2000; Yu et al., 2010). It is likely that the differences observed in PML-NB structure of PML-NBs formed by the individual isoforms are due, at least in part, to the differences in interacting proteins recruited to PML-NBs formed by the different isoforms. The difference in the SUMO2/3 modification state of each PML isoform is also likely to influence the structure of PML-NBs.

We used high-content imaging to quantify EYFP-PML fluorescence for each isoform after arsenic treatment. PML accumulates in large PML-NBs after arsenic treatment in the absence of RNF4, the ubiquitin E3 ligase required for arsenic-induced degradation of PML (Geoffroy et al., 2010; Lallemand-Breitenbach et al., 2008; Tatham et al., 2008). We therefore developed an assay for the size of PML-NBs as a measure of this response, and also to assess the size of any cytoplasmic inclusions of PML. This confirmed the differences in susceptibility of the different isoforms to arsenic treatment, with PML IV being the least degraded after 24 hours of treatment (Fig. 3). The localisation of PML following arsenic treatment also differed between isoforms, with PMLI, PMLII and PMLVI accumulating in the cytoplasm after prolonged arsenic treatment (Figs 2,3). A recent report (Lång et al., 2012) described the formation of cytoplasmic accumulations of PML and nucleoporins (CyPNs) following arsenic treatment in cells overexpressing PMLI, both on a background of endogenous PML and in cells depleted of PML. Here, we identify formation of PML cytoplasmic inclusions for three of the six PML isoforms examined (Figs 2, 3). Although we have not investigated nucleoporin colocalisation in this study, PMLI, PMLII and PMLVI found in cytoplasmic foci following arsenic treatment did not colocalise with SUMO2/3 and therefore were unlikely to be SUMO modified (Fig. 2). This was described for CyPNs reported previously (Jul-Larsen et al., 2009; Lång et al., 2012). The sequence of PMLVI is common to all isoforms, although it lacks the SIM that is present in all others (Jensen et al., 2001). It is therefore interesting that it localises differently to PMLIII, PMLIV and PMLV in response to arsenic treatment. This suggests that elements encoded by the C-terminal regions of PMLIII, PMLIV and PMLV preclude CyPN formation, whereas the C-terminal regions of PMLI and PMLII do not.

The ubiquitin E3 ligase RNF4 is required for degradation of all six PML isoforms because siRNA-mediated depletion of RNF4 prior to arsenic treatment resulted in the accumulation of high molecular weight PML species, which were found to be located within PML-NBs by fluorescence microscopy (Fig. 6). This was also apparent from a previously published report (Geoffroy et al., 2010) in which the PML degradation response to arsenic was shown to be blocked by prior treatment of cells with cycloheximide. However, this observation was explained by the rapid depletion of RNF4 under conditions of protein synthesis inhibition. An intriguing finding of this work was the striking effect of RNF4 depletion in cells expressing PMLV. It has previously been reported that RNF4 ubiquitylates PML under basal conditions, because an increase in PML expression is identified following RNF4 depletion (Tatham et al., 2008). Although there is a slight increase in expression of PMLIII following RNF4 depletion, there was marked accumulation of high molecular weight PMLV species, which were increased yet further following arsenic treatment (Fig. 6). This suggests that PMLV is a particularly good substrate for RNF4, and might account for the low levels of PMLV observed in this (Fig. 1C) and other studies (Cuchet et al., 2011). This is also consistent with the observation that PMLV is the PML isoform most readily degraded in response to arsenic treatment (Fig. 1D; Figs 2, 3). RNF4 is recruited to PML modified with polymeric SUMO chains in PML-NBs following arsenic treatment through four SUMO-interaction motifs in the N-terminal region of the protein (Geoffroy et al., 2010; Lallemand-Breitenbach et al., 2008; Tatham et al., 2008). That PMLV is a particularly good substrate for RNF4 suggests that PMLV might be the most highly SUMO-modified isoform. This hypothesis is supported by both western blot and immunoprecipitation analysis (Fig. 1C; Fig. 5). PMLV has previously been suggested to act as a scaffold in PML-NBs, because it is particularly stable in PML-NBs when analysed by fluorescence recovery after photobleaching (FRAP) (Brand et al., 2010; Weidtkamp-Peters et al., 2008). PMLV was also demonstrated to form large PML-NBs when expressed in PML^−/−^ cells (Condemine et al., 2006). PML mutants, which cannot be sumoylated on K160 or K490, display very fast FRAP kinetics at PML-NBs (Weidtkamp-Peters et al., 2008). The increased residence times observed for PMLV might therefore be due to its high levels of SUMO modification at one or both of these residues. PMLV is one of only two PML isoforms conserved from mouse to humans (Condemine et al., 2006), which suggests that functions dependent on the unique C-terminal region of PMLV might be particularly important, perhaps including this potential function as a scaffold component for PML-NBs. The importance of this region of PMLV is further supported by the observation that the C-terminal region of PMLV is capable of forming punctate nuclear structures in the absence of other PML isoforms, and recruits other PML-NB components to these structures (Geng et al., 2012).

In response to arsenic, 3D structured illumination analysis revealed dramatic and isoform-specific changes to PML body ultrastructure. PMLI, PMLII and PMLVI accumulate in the cytoplasm, whereas PMLIII, PMLIV and PMLV remain associated with the nucleus. PMLIV was identified as the isoform most resistant to degradation whereas PMLV was the isoform most readily degraded following arsenic treatment. Depletion of RNF4, using siRNA, resulted in marked accumulation of PMLV, suggesting this isoform is an optimal substrate for RNF4. This study indicates that the variable C-terminal region of PML, generated by alternative splicing, plays a crucial role in the susceptibility of the distinct PML isoforms to arsenic-induced degradation.

## MATERIALS AND METHODS

### Cell culture and arsenic treatment

HepaRG hepatocytes expressing enhanced yellow fluorescent protein (EYFP)-tagged PML isoforms were cultured in Williams Medium E (Gibco), with 10% fetal bovine serum gold (PAA), 2 mM glutamine, 5 µg/ml insulin, 0.5 µM hydrocortisone and 100 U/ml penicillin and streptomycin. Cells were maintained under antibiotic selection with G418 and puromycin. Parental HepaRG hepatocytes were cultured under the same conditions, without antibiotic selection. NB4 cells were cultured in RPMI 1640 medium (Gibco) supplemented with 2 mM glutamine and 100 U/ml penicillin and streptomycin. Cells were cultured for 24 hours before arsenic treatment was commenced. Culture medium was replaced with medium containing arsenic trioxide stock solution (1 mM in Tris-buffered saline), diluted to a final concentration of 1 µM.

### Western blotting

HepaRG cells were washed twice in PBS, lysed in 2× SDS lysis buffer (150 mM Tris-HCl, pH 6.8, 25% glycerol, 5% SDS, 0.01% Bromophenol Blue), sonicated briefly to shear DNA and protein estimation performed using the DC protein estimation kit (Bio-Rad). NB4 cells were lysed in 8 M Urea with 0.1 M dithiothreitol, sonicated briefly and protein concentration estimated using the Bio-Rad protein assay. Equal amounts of total protein were then resolved by SDS-PAGE using 4–12% Bis-Tris gels, and transferred to PVDF membrane for immunoblotting. Membranes were blocked in 5% non-fat milk in 0.1% Tween-20/PBS for 30 minutes prior to incubation with primary antibody in 5% non-fat milk in 0.1% Tween-20/PBS for 1 hour at room temperature. Following three washes with 0.1% Tween-20/PBS, membranes were incubated with appropriate secondary antibodies in 5% non-fat milk in 0.1% Tween-20/PBS for 1 hour at room temperature, washed a further three times and processed using enhanced chemiluminescence.

### Antibodies

Affinity-purified chicken anti-PML, chicken anti-RNF4, sheep anti-SUMO1 and sheep anti-SUMO2/3 antibodies were generated in house. Anti-ubiquitin (DAKO), anti-β-actin (Sigma), and anti-tubulin (Amersham) antibodies were purchased from commercial sources. Rabbit anti-RARA 115 was a kind gift from Cecile Egly (IGBMC, Strasbourg, France). Horseradish-peroxidase-conjugated secondary antibodies against chicken, sheep, rabbit and mouse IgG were purchased from Sigma. Secondary antibodies used for immunofluorescence, Cy-5-conjugated and Dylight-594-conjugated anti-sheep IgG were purchased from Jackson Immunochemicals.

### Immunofluorescence

Cells were cultured on coverslips, washed twice in PBS and then fixed with 4% paraformaldehyde in PBS for 10 minutes at 37°C degrees. Cells were permeabilised with 0.2% Triton X-100 in PBS, blocked in 5% BSA, 0.1% Tween-20 in PBS for 30 minutes at room temperature and incubated in primary then secondary antibodies diluted in 1% BSA, 0.1% Tween-20 in PBS for 1 hour each, also at room temperature. Cells were then stained with 0.1 µg/ml DAPI and mounted using Vectashield mounting medium. Images were collected using a Deltavision DV3 microscope and processed using Softworx software (Applied precision). Images are presented as maximal intensity projections of multiple z-sections.

### High-content imaging

Cells were cultured in black, clear-bottomed 96-well plates (Corning CellBIND) in 100 µl culture medium for 24 hours. For arsenic treatment, 10 µl of 11 µM arsenic trioxide diluted in culture medium was added to wells using a multichannel pipette, to give a final concentration of 1 µM. At the desired time points, cells were washed twice with PBS, fixed with 4% paraformaldehyde, permeabilised and stained with DAPI as described above. 100 µl of PBS was dispensed into wells and plates were thermally sealed using an X-Seal Manual Thermal Sealer (Fluid X) ready for imaging.

Imaging was performed using an IN Cell 2000 microscope (GE Healthcare) to acquire two fields of view per well with a 20× lens (Nikon), capturing DAPI and EYFP fluorescence using 350 nm and 500 nm light sources respectively. Images were analysed using IN Cell Investigator software (GE Healthcare), using a protocol designed to identify PML inclusions by multi-scale top hat transformation. The measure of total area of PML per cell nucleus or cytoplasm was selected as the most discriminatory for changes in PML following arsenic treatment. Data were obtained for >5000 cells per condition and averaged to give a value for each isoform under three conditions, 0, 1 and 24 hours of arsenic treatment. Data were then normalised to the value for 0 hours of arsenic treatment for each isoform, for ease of comparison across isoforms. Data presented represent the mean ± s.e.m.

### Structured illumination

Samples were prepared as for immunofluorescence prior to imaging using the OMX version 2 system (Applied Precision) as previously described (Hattersley et al., 2011). Images were acquired using a 100×, 1.4 NA, oil-immersion objective lens (Olympus, Center Valley, PA) and back-illuminated Cascade II 512×512 electron-multiplying charge-coupled device (EMCCD) camera (Photometrics, Tucson, AZ) on the OMX version 2 system (Applied Precision) equipped with 405, 488 and 593 nm solid-state lasers. Samples were illuminated by a coherent scrambled laser light source that had passed through a diffraction grating to generate the structured illumination by interference of light orders in the image plane to create a 3D sinusoidal pattern, with lateral stripes ∼0.2 µm apart. The pattern was shifted laterally through five phases and through three angular rotations of 60′ for each Z-section, separated by 0.125 µm. Exposure times were typically between 200 and 500 ms, and the power of each laser was adjusted to achieve optimal intensities of between 2000 and 4000 counts in a raw image of 16-bit dynamic range, at the lowest possible laser power to minimize photo bleaching. Raw images were processed and reconstructed to reveal structures with greater resolution. The channels were then aligned in *x*, *y*, and rotationally using predetermined shifts as measured using a target lens and the Softworx alignment tool (Applied Precision).

### Immunoprecipitation

HepaRG cells expressing PML isoforms were cultured in 10 cm plates and treated with arsenic as described above prior to harvesting by scraping after two washes with PBS containing 100 mM iodoacetamide on ice. Cells were pelleted by centrifugation at 400 ***g*** and lysed in ice-cold RIPA buffer (50 mM Tris-HCl, pH 7.5, 150 mM NaCl, 1% NP-40, 0.5% deoxycholate) and 100 mM iodoacetamide with end-over-end rotation for 20 minutes at 4°C. Lysates were clarified by centrifugation at 17 000 ***g*** for 10 minutes and precleared by incubation with Sepharose beads for 1 hour, followed by overnight incubation with agarose beads coupled to a single-chain, recombinant GFP antibody (a gift from the Division of Signal Transduction Therapy, University of Dundee) with constant end-over-end mixing at 4°C. Beads were then washed three times with RIPA buffer and bound proteins eluted in 2× SDS lysis buffer, and analysed by SDS-PAGE and immunoblotting.

### siRNA transfections

Cells were transfected with a pool containing an equal amount of four siRNA duplexes targeting RNF4 (Dharmacon ON-TARGET plus; RNF4, 1-GCUAAUACUUGCCCAACUUUU; RNF4, 2-GAAUGGACGUCUCAUCGUUUU; RNF4, 3-GACAGAGACGUAUAUGUGAUU; RNF4, 4-GCAAUAAAUUCUAGACAAGUU) to a final concentration of 10 nM, or a non-targeting control duplex at the same concentration using Lipofectamine RNAiMAX (Invitrogen) according to the manufacturer's instructions. Arsenic treatment was commenced 48 hours after transfection.

For high-content imaging of RNF4-depleted cells, cells were reverse transfected with the RNF4 siRNA pool described above or a non-targeting control duplex in 96-well plates, with a final siRNA concentration of 10 nM. 10 µl of 100 nM siRNA was dispensed into wells, followed by 10 µl of a 1∶50 dilution of RNAiMAX/opti-MEM (Invitrogen) serum-free medium mix. This was incubated for 15 minutes at room temperature prior to the addition of 5000 cells in 80 µl of antibiotic-free culture medium per well. Arsenic treatment was commenced at 48 hours after transfection, and cells were fixed, stained, imaged and analysed as described above.

## Supplementary Material

Supplementary Material
