# Supplementary material for: PML isoforms in response to arsenic: high-resolution analysis of PML body structure and degradation
Source: J Cell Sci. 2014 Jan 15;127(2):365–75. doi: 10.1242/jcs.132290 (PMC3889398; doi:10.1242/jcs.132290)
Supplement: Supplementary Material [file supp_127_2_365__index.html]

Supplementary Material 

# PML isoforms in response to arsenic: high-resolution analysis of PML body structure and degradation

## JCS132290 Supplementary Material

**Files in this Data Supplement:**

- **Supplementary Material PDF**
