# Supplementary material for: PML isoforms in response to arsenic: high-resolution analysis of PML body structure and degradation
Source: J Cell Sci. 2014 Jan 15;127(2):365–75. doi: 10.1242/jcs.132290 (PMC3889398; doi:10.1242/jcs.132290)
Supplement: Supplementary Material [file supp_jcs.132290_JCS132290.pdf]

A

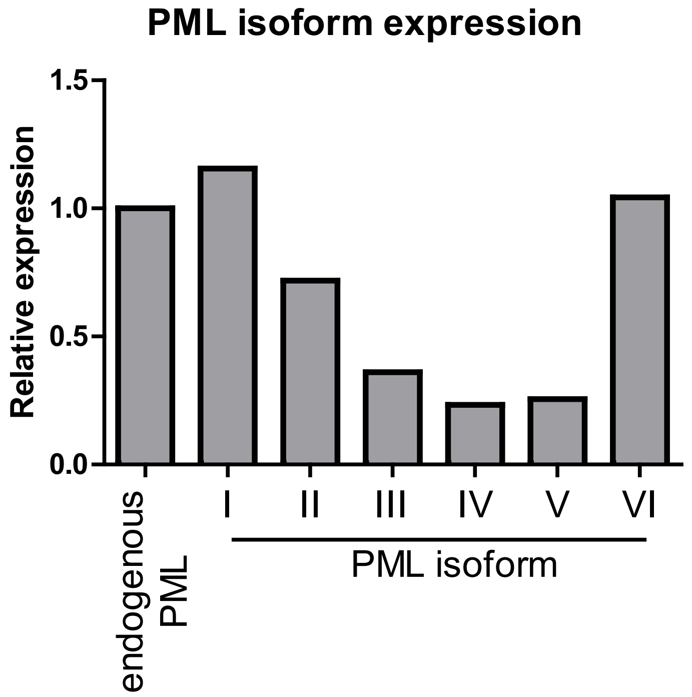

B

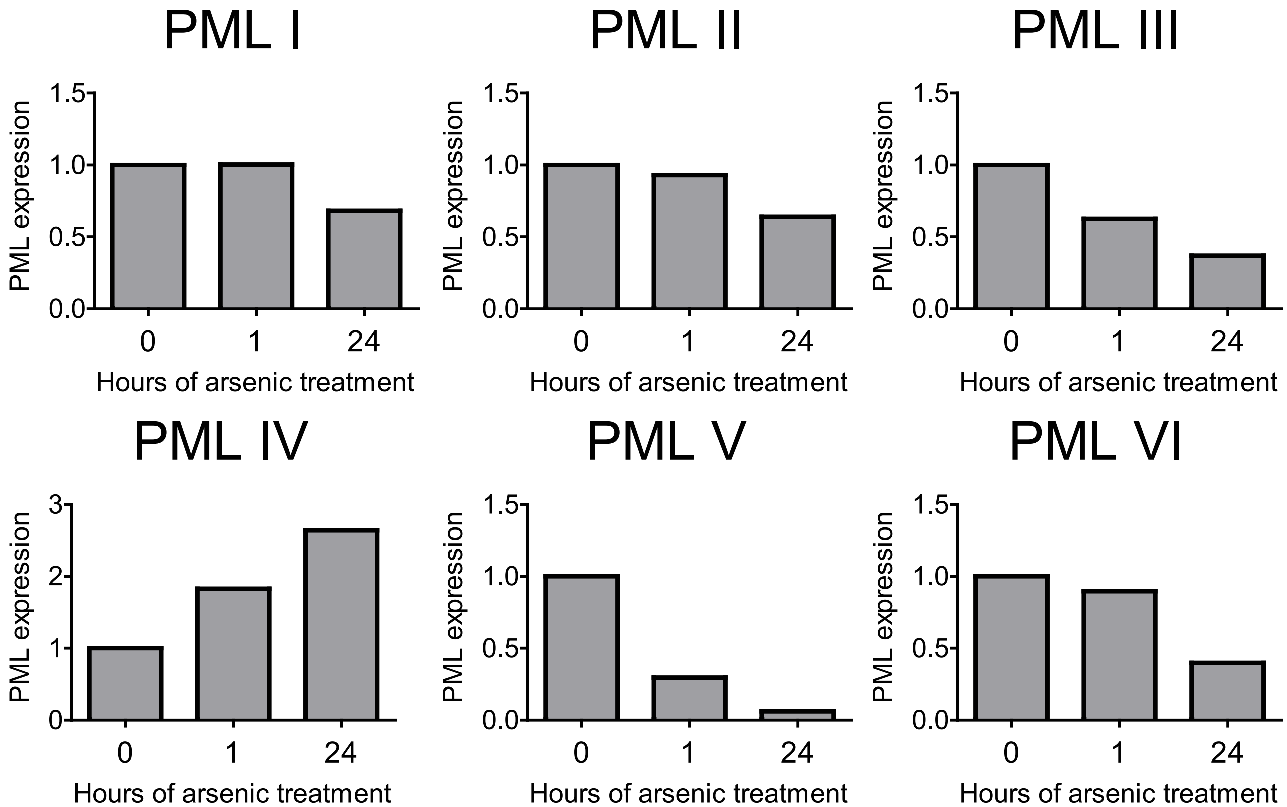

Fig. S1. (A) Quantification of endogenous PML (HALL cells) and stably expressed EYFP-PML isoforms (Fig. 1C) was performed by densitometric analysis using ImageJ software. Data were normalised to endogenous PML expressed in HALL cells. (B) Quantitation of EYFP-PML isoform expression before and after arsenic treatment (Fig. 1D) was performed by densitometric analysis using ImageJ software. Data were normalised to isoform expression at 0 hour time point.
